# Relevance of Religiosity for Coping Strategies and Disability in Patients with Fibromyalgia Syndrome

**DOI:** 10.1007/s10943-020-01177-3

**Published:** 2021-01-23

**Authors:** Alexandra Braun, Dimitar Evdokimov, Johanna Frank, Paul Pauli, Thomas Wabel, Nurcan Üçeyler, Claudia Sommer

**Affiliations:** 1 Department of Neurology, University of Würzburg, Josef-Schneider-Straße 11, 97080 Würzburg, Germany; 2 Department of Psychology (Biological Psychology, Clinical Psychology and Psychotherapy), Center of Mental Health, University of Würzburg, Marcusstraße 9-11, 97070 Würzburg, Germany; 3 Department of Systematic Theology, University of Bamberg, Markusplatz 3, 96047 Bamberg, Germany

**Keywords:** Fibromyalgia syndrome, Religiosity, Coping, Disability

## Abstract

Coping strategies are essential for the outcome of chronic pain. This study evaluated religiosity in a cohort of patients with fibromyalgia syndrome (FMS), its effect on pain and other symptoms, on coping and FMS-related disability. A total of 102 FMS patients were recruited who filled in questionnaires, a subgroup of 42 patients participated in a face-to-face interview, and data were evaluated by correlation and regression analyses. Few patients were traditionally religious, but the majority believed in a higher existence and described their spirituality as “transcendence conviction”. The coping strategy “praying–hoping” and the ASP dimension “religious orientation” (*r* = 0.5, *P* < 0.05) showed a significant relationship independent of the grade of religiosity (*P* < 0.05). A high grade of belief in a higher existence was negatively associated with the choice of ignoring as coping strategy (*r* = − 0.4, *P* < 0.05). Mood and affect-related variables had the highest impact on disability (*b* = 0.5, *P* < 0.05). In this cohort, the grade of religiosity played a role in the choice of coping strategies, but had no effects on health and mood outcome.

**Supplementary Information:**

The online version of this article (10.1007/s10943-020-01177-3) contains supplementary material, which is available to authorized users.

## Introduction

Fibromyalgia syndrome (FMS) is an incompletely understood chronic pain condition accompanied by symptoms like sleep disturbance, depression or fatigue (Clauw 2015; Clauw et al. 2011; Häuser et al. 2015). Pathophysiological mechanisms in the central and peripheral nervous system as well as psychological factors have been shown to play a role (Afari et al. 2014; Clauw 2015; Leinders et al. 2016; Paiva et al. 2008; Park and Lee 2017; Sluka and Clauw 2016; Staud and Smitherman 2002; Üçeyler et al. 2013; Zimmermann 1991). There is large heterogeneity of patient profiles in FMS patients, in particular regarding coping strategies (Alok et al. 2014; Kengen-Traska et al. 2012; Loevinger et al. 2012; Stoffel et al. 2013; Tommaso et al. 2014; Triñanes et al. 2014; Yim et al. 2017). Some patients cope very well with the symptoms, while others are heavily affected in their daily life with passive and negative coping styles like catastrophizing, ignoring, and helplessness (Baastrup et al. 2016).

A large body of evidence shows that religious involvement is related to better psychological well-being, enhanced social support, less depression, and reduced substance abuse (Baetz and Bowen 2008; Basiński et al. 2013; Büssing et al. 2005, 2009, 2010, 2014, 2013 ; Dedert et al. 2004; Dezutter et al. 2009; Kendler et al. 1997; Mishra et al. 2017; Wachholtz et al. 2007). Only few studies have analyzed the influence of religiosity on pain sensitivity or intensity in chronic pain patients (Basiński et al. 2013; Fehring et al. 1997; Gilbert 2009), and even less so in FMS (Anema et al. 2009; Biccheri et al. 2016; Moreira-Almeida and Koenig 2008). The evaluation of the impact of religiosity is difficult because of the variety of possible definitions of religiosity. Religiosity is a multidimensional construct including cognition, feelings, and behavior with institutional affiliation (Huber 2003; Koenig et al. 2001). Beside this definition there are others, but all of them are commonly based on religious practice and doctrine as opposed to spirituality (Park et al. 2013). The definition of spirituality which we follow in this study was given 2009 by the International Consensus Conference as “aspects of humanity that refer to the way individuals seek and express meaning and purpose and the way they experience their connectedness to the moment, to self, to others, to nature, and to the significant or sacred” (Puchalski et al. 2009). Whether religiosity and spirituality might have a positive impact on health and on coping with disease has been discussed controversially (Berthold and Ruch 2014; Klein and Albani 2007; Koenig 2012; Reis and Menezes 2017; Wachholtz et al. 2007). Believing and hoping are important factors that may improve mental strength in aversive situations (Berthold and Ruch 2014; Nejat et al. 2017; Paloutzian and Park 2013), and the relationship to God as an abstract social support might replace lacking family connections (Anson et al. 1990; McIntosh et al. 1993) and might function as a strong resilience-driving element.

In this study, we first examined whether FMS patients have specific spiritual or religious needs, and whether they use religious strategies to cope with emotional aspects of FMS and every day pain. This was achieved by assessing religiosity with specific questionnaires and in a subgroup of patients with an additional semi-structured interview. We then asked whether religiosity is helpful or disadvantageous for coping with FMS and which would be the impact of different aspects of religiosity on FMS-related disability. In the long-run, our findings might contribute to improving therapies by considering the religiosity of patients.

## Methods

### Study Participants

This study is part of a currently larger study on FMS at the Department of Neurology of the University Hospital Würzburg that included 148 FMS patients (Wolfe et al. 2010) and 46 healthy controls who were recruited between 2015 and 2018. Inclusion and exclusion criteria were published elsewhere (Üçeyler et al. 2013). Our study was approved by the Würzburg Medical School Ethics Committee (No. 135/15). All patients provided written informed consent before enrollment. Data on clinical examination, electrophysiological, and other laboratory measurements will be published elsewhere.

### Questionnaires used for Correlation and Regression Analyses

All study participants filled in a set of standardized questionnaires collecting data on religiosity and spirituality (Bϋssing et al. 2007), coping strategies (Verra et al. 2006), pain (Sommer et al. 2011; Türp and Nilges 2000), depression (Meyer and Hautzinger 2001), anxiety (Laux et al. 1981), and quality of life due to FMS (Offenbaecher et al. 2000). Because some questionnaires were added during the ongoing study, we present data for 102 patients (Supplementary Fig. 1).

### Convictions and Attitudes Related to the Dimension of Religiosity/Spirituality

The Aspects of Spirituality (ASP) questionnaire examines a wide range of aspects of spirituality on a scale from 0 to 4 with “does not apply at all” to “applies very much”. Four subscales reflect dimensions such as religious orientation, search for insight/wisdom, conscious interactions and transcendence conviction. “Religious orientation” includes traditional religious activities like praying to God or attending religious services. “Transcendence conviction” indicates spirituality, belief in the existence of higher realities and rebirth. The dimension “search for insight/wisdom” includes a mindful spirit of broad awareness and developing wisdom. The dimension “conscious interactions” describes compassion and generosity. Subgroups of each dimension are defined as low grade, i.e., sum scores < 50 and as high grade, i.e., sum scores ≥ 50 (Bϋssing et al. 2007).

### Psychological Interview

A subgroup of 42 patients additionally participated in a semi-structured 20-minute face-to-face interview consisting of three parts: early life stress (part 1), parameters of religiosity including morality (part 2), and parameters of problem-solving behavior including learning (part 3; supplementary Fig. 2). The protocol contained elements of the Life History Calendar (Freedman et al. 1988), questions on morality and religion adapted on an interview form developed by the Department of Psychology of the University of Würzburg and self-developed items to evaluate problem solving and learning behavior. These items are known resilience promoting psychological factors in literature (Navrady et al. 2018; Niitsu et al. 2018; Rutter 1985; Treichler et al. 2019).

### Coping Strategies

The Coping Strategies Questionnaire (CSQ) consists of 8 subscales (distraction of attention, reinterpretation, self-instructions, ignoring, praying and hoping, catastrophizing, increase in activity, pain behavior) on a 1 to 6 scale ranging from “never” to “always” and two sum items indicating self efficacy. The maximum possible value for each coping strategy sum score is 36 (Verra et al. 2006).

### Depression, Pain Catastrophizing, and Anxiety

The German Version of the Center of Epidemiological Studies General Depression Scale (CES-D) examines the severity of depressive symptoms on a 0 to 3 scale from “rare” to “mostly”. A score of CES-D ≥ 16 indicates depressive symptoms that may be of clinical relevance (Meyer and Hautzinger 2001). The German version of the Pain Catastrophizing Scale (PCS) uses 13 items to examine the strength of catastrophizing thoughts and behavior on a 0 to 4 scale from “never” to “always” (Meyer et al. 2008). We used the State-Trait Anxiety Inventory (STAI-G) to examine anxiety as a trait (STAI-T) and as a state (STAI-S) on a 1 to 4 scale from “almost never” to “almost always” (Laux et al. 1981).

### Pain and FMS Questionnaires

The Graded Chronic Pain Scale (GCPS) examines the current severity of pain, pain in the last six months with influence on daily activities, free time, and job on a 0 to 10 scale from “no disability” to “no activity possible”. A grade of disability was calculated (Türp and Nilges 2000). To assess neuropathic pain components, the German Version of the Neuropathic Pain Scale Inventory (NPSI-D) was used (Sommer et al. 2011). FMS symptom severity and impact were measured by the Fibromyalgia Impact Questionnaire (FIQ) that examines physical and emotional functioning and related disability of FMS patients (Offenbaecher et al. 2000).

### Study Design

One week before the appointment, all questionnaires were sent to the patients and were brought back filled in on the study day. After a detailed history taking that collected demographic data, starting date of FMS, and family history, the psychological interview was conducted.

### Statistical Analysis

SPSS Statistics 24 software (IBM, Ehningen, Germany) was used for statistical analysis. Data distribution was tested with the Shapiro–Wilk test and by observing data histograms. Results of the non-normally distributed data are given as median and range, normally distributed data are given as mean and standard deviation. Differences in mean scores were tested by the Wilcoxon test for nonparametric-related samples. Correlation was analyzed by the Spearman correlation coefficient to select potentially relevant variables for regression analysis and p values were corrected with Benjamini–Hochberg correction. For subgroup analyses on religiosity and the preference for a specific coping strategy, we applied the Chi-square test. A hierarchical multiple regression analysis was conducted to determine the contribution of variables and coping strategies in the outcome variable “FMS impact in life”. *P* < 0.05 was considered significant.

## Results

### Demographic Characteristics

Demographic characteristics of 102 study participants (96 women) are listed in Table 1. On average, patients had suffered from FMS for 14.71 years in median. A total of 47 out of 102 (47.94%) patients had a family history of pain-related diseases and 85/102 (86.70%) patients of affective mood disorders. A total of 50 out of 102 (51%) patients had experienced a traumatic life event which was, for example, the death of a close relative or sexual, physical or emotional abuse and neglect. Among the 42 patients who participated in the psychological interview, 17/42 (7.14%) patients were catholic, 13/42 (5.46%) protestant, 1/42 (0.42%) muslim, and 11/42 (4.62%) without any confession (Table 1).

**Table 1:** Sociodemographic characteristics

Variables	*N*
Sample size	102
Age^a^	50.5 ± 53.2
Female/male	96/6
Ø Weight^b^	75.0 ± 15
Height^c^	166.3 ± 7.4
BMI	25.3 ± 5.1
Disease duration^d^	14.7 ± 11.2
Highest level of education^e^	
University diploma	14
A-level	14
0-level	55
Secondary school only	19
Current employment status	
Regularly working	57
Sick leave all/sick leave because of pain	15
Retired all/retired because of pain	23
Unemployed	5
Psychological/psychiatric treatment	
Never	40
Currently	39
In the past	23
Family history of diseases	
Chronic pain	47
Neurological disorder	84
Affective disorders	85
Life Event	
Yes	50
No	52
Confession (*N* = 42)	
Catholic	17
Protestant	13
Islamic	1
None	11

*N* number

^a^Year (in median and range)

^b^Kilogram in mean ± SD

^c^Centimeter in mean ± SD

^d^Year (in mean ± SD)

^e^Highest level of education: A-level: High school diploma allowing university access (12–13 years of school), O-level: O-level diploma after 10 years of school, Secondary school only: lower secondary school diploma after 8–9 years of school

### Religiosity and Spirituality According to the Four Dimensions of the ASP Questionnaire

**Fig. 1:**
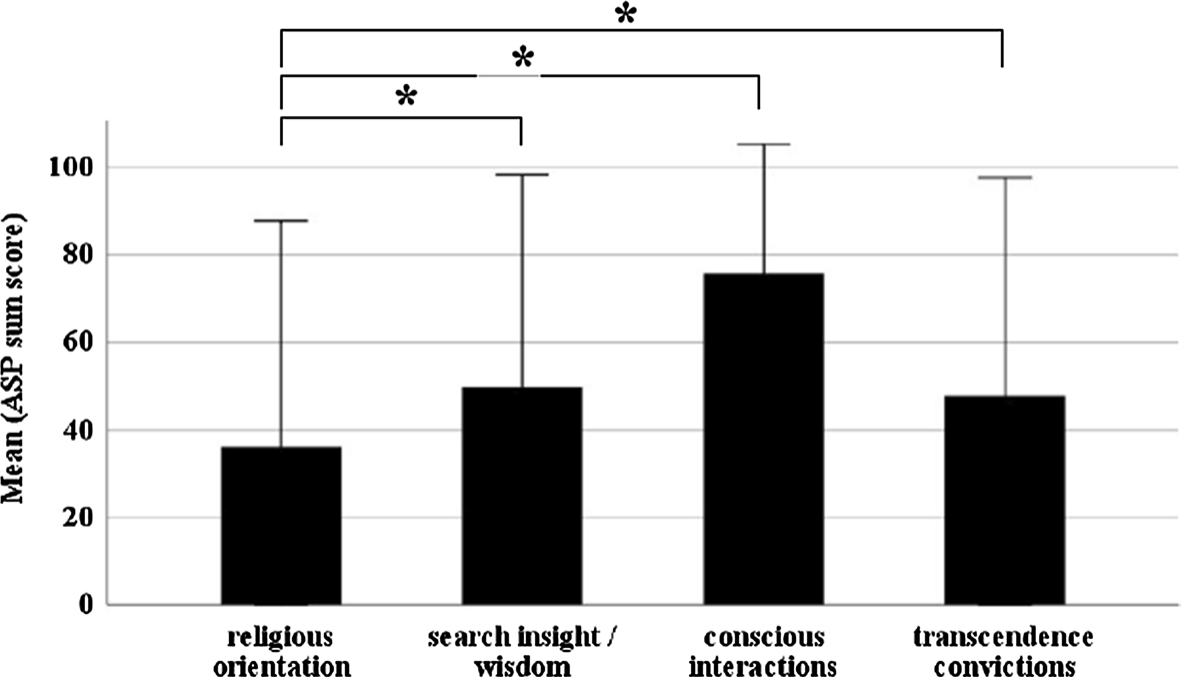
Religiosity according to the ASP questionnaire. Bar graphs show mean scores of all four religious’ dimensions. The first dimension “religious orientation” (*M* = 36.2, SD = 25.8) shows a significantly lower mean score than conscious interactions (75.8 ± 14.7, *P* < 0.001). This combined with high values for “transcendence convictions” (*M* = 47.7, SD = 25.0, *P* < 0.001), suggests a more spiritual than traditionally religious patient cohort

We used the ASP questionnaire (maximum sum scores for each dimension 100) to evaluate the religiosity in our patient cohort and to differentiate between the four dimensions of religiosity and spirituality. Patients on average reported relatively low scores in the dimension “religious orientation” (36.2 ± 25.8, Fig. 1) and higher scores in the dimension “conscious interactions” (75.8 ± 14.7; *z* = − 8.8, *P* < 0.001, *r* = − 0.8, Wilcoxon test). However, we have no reference values but this outcome suggests that most patients frequently interact in a conscious way with the environment and other people. The mean scores for (1) “search of insight/wisdom” and (2) “transcendence conviction” were 49.7 ± 24.3 and 47.7 ± 25.0, respectively, and both significantly differed from religious orientation (z_(1)_ = − 4.9, *P* < 0.001, *r* = − 0.5; z_(2)_ = − 4.6, *P* < 0.001, *r* = − 0.4; Fig. 1).

### Religiosity and Spirituality According to the Interview

During the psychological interview, patients were asked to characterize their religious preferences (supplementary Table 1). Five patients defined their believing type as “spiritual”, 9/42 (21%) as “religious”, 4/42 (10%) as “atheistic”, 1/42 (2%) as “agnostic” and 1/42 (2%) as “not determined”, but 22/42 (52%) defined their belief with their own words. This comes as no surprise, given the highly subjective character of religious and spiritual self-description (Eisenmann et al. 2016). In order to operationalize this heterogeneity, we decided to use only the parameter “belief in a higher existence” with grading from “none” to “intense” (Supplementary Table 2), knowing that this means a certain degree of simplification. 27/42 (11.34%) patients had moderate to strong beliefs in a higher existence, while only 15/42 (6.30%) had none to low belief.

### Influence of Religiosity on Coping and Health Outcomes

Spearman correlations were applied to assess associations between religiosity and demographic variables, health outcomes and coping strategies. All coefficients had a low or moderate strength, so that no clear conclusion can be reached; however, these values provide information and hints about suspected connections. The correlation between the coping strategy “praying—hoping” and the ASP dimensions of “religious orientation” (*r* = 0.5, *P* < 0.05) might suggest a higher use of “religious coping” in those with higher scores in the religious and spiritual dimensions, as expected (Table 2). After Benjamini–Hochberg correction the correlation between coping “praying-hoping” and “transcendence conviction” (*r* = 0.3, *P* < 0.05) remained only a trend.

**Table 2:** Correlation between ASP dimensions and predicting variables

Predicting variable	ASP dimension	*r*	*p*	Corrected *p*
Distraction	Transcendence convictions	0.268	0.007	0.149
	Search insight wisdom	0.210	0.037	0.4736
Praying hoping	Religious orientation	0.476	0.000	0.000*
	Search insight wisdom	0.208	0.039	0.416
	Transcendence convictions	0.306	0.002	0.064
Pain duration	Religious orientation	–	–	
GCPS grade	Search insight wisdom	0.225	0.025	0.4

*R* Spearman‘s Rho, correlation coefficient

*ASP* aspects of spirituality, *GCPS* graded chronic pain scale

*Correlation is significant at the 0.05 level (2-tailed) after Benjamini–Hochberg correction

To examine whether the patients used a specific coping strategy related to their grade of belief in a higher existence, we created two subgroups with high or low agreement for each of the four ASP dimensions performed a Chi-square test. Patients with high or low agreement for any of the ASP dimensions did not differ in their coping strategies. However, when a Chi-square test was done with the subgroups of “none to low” versus “moderate to intense” religiosity as derived from the interview, we found that patients with higher belief in a higher existence preferred the coping strategies “ignore”, “catastrophizing” and “pain behavior” (*P* < 0.05).

### Influence of Psychological, Physical, Spiritual and Demographic Variables on FMS-Related Disability

A hierarchical multiple regression analysis was conducted to analyze whether some FMS-related variables, choice of coping type or religiosity had an influence on the impact of FMS on daily life. The sum score of the FIQ (Offenbaecher et al. 2000) was set as dependent variable to indicate FMS-related disability. The hierarchical multiple regression model contains five models with gradually added variables (supplementary Table 3). The results of 99/102 (97%) patients were valid to be involved into the regression analysis.

Model one, which included demographic variables, explained only 2.7% of the variance, indicating minor importance to the impact of FMS on daily life (Table 3). Model two, in which three pain-related variables were added, explained 38.4% of the variance. In model three, the *R*^2^ was doubled after adding variables of psychopathology. By adding the variables depression, anxiety (trait/state) and pain catastrophizing, 61.6% of the variance could be explained. This shows that variables related to affect and mood have a high impact on disability. After adding coping variables to the model, the variance explaining *R*^2^ was increased to 66%. Finally, after adding the four dimensions of religiosity to the model, the squared R minimally increased (67.1%), which reflects the lower importance of any dimension of religiosity for disability in our cohort. The entire model showed a moderate cross validity (Durbin Watson = 1.7) (Table 3). The F ratio of every model (except model 1) showed a value > 1, which indicates a significant improvement of predicting the outcome (FMS impact in life) compared to not fitting the model (supplementary Table 4).

**Table 3:** The summary of the regression model and the variance explained by every added predictor variables

Model^a^	*R*^2^	Adjusted *R*^2^	*R*^2^ change	F change	Significant F change	Durbin Watson
1	0.027	− 0.0	0.0	0.7	0.6	
2	0.384	0.3	0.4	17.6	0.0	
3	0.616	0.6	0.2	13.2	0.0	
4	0.660	0.6	0.0	1.0	0.5	
5	0.710	0.6	0.0	0.6	0.7	1.7

^a^Dependent variable: FMS impact in life; squared R: *R*^2^ shows how much of the variability in the outcome is accounted for by the predictors

The b-values give a measure of the individual contribution of each predictor to the final model (Table 4). Positive b-values indicate a positive relationship between the outcome and the predictor. The variables “reinterpretation” as coping, “pain intensity”, “GCPS grade” and “depression” made a significant contribution (*P* < 0.05) to the model with the largest impact made by the variable “depression” (*t* = 3.2, *P* < 0.05). All four dimensions of religiosity had little influence on the model, as well as most of the coping strategies except reinterpretation and two pain variables. When we also looked at the standardized b-values (β-values), we could see the same effect for especially the variable “depression” (Table 4).

**Table 4:** Individual contribution of each predictor to the chosen model five and the relationship between the FMS impact in life and each parameter

Category	Predictor variable^a^	Unstandardized b	Standardized β	*T*	Significance
Constant		**36.9**		**1.4**	**0.2**
**Demographic variable**	Age	− 0.0	− 0.0	0.1	0.9
	Height	− 0.1	0.1	− 0.7	0.5
	BMI	0.0	0.0	0.1	0.9
	Pain duration	0.1	0.1	0.7	0.5
**Pain variables**	Neuropathic pain	2.4	0.0	0.4	0.7
	**Pain intensity**	**0.2**	**0.2**	**2.0**	**0.0**
	**GCPS grade**	**4.0**	**0.2**	**2.4**	**0.0**
**Psycho-pathological variables**	Pain catastrophizing	− 0.2	− 0.2	1.1	0.3
	**Depression**	**0.5**	**0.4**	**3.2**	**0.0**
	State anxiety	− 0.0	− 0.0	− 0.3	0.8
	Trait anxiety	0.2	0.2	1.0	0.3
**Coping strategies**	Distraction	− 0.0	− 0.0	− 0.1	1.0
	**Reinterpretation**	**0.3**	**0.2**	**2.1**	**0.0**
	Self-instructions	− 0.2	− 0.1	− 1.0	0.3
	Ignore	0.2	0.1	1.0	0.4
	Praying hoping	0.1	0.1	1.0	0.5
	Catastrophizing	0.1	0.1	0.5	0.6
	Activity increase	− 0.2	− 0.1	− 1.0	0.3
	Pain behavior	− 0.1	− 0.0	− 0.4	0.7
**Dimension of religiosity**	Religious orientation	− 0.0	0.0	0.1	0.9
	Search insight/wisdom	0.0	0.0	0.2	0.8
	Conscious interaction	− 0.1	− 0.1	− 1.4	0.2
	Transcendence convictions	0.0	0.1	0.6	0.6

Bold values are statistically significant (Data significance at p < 0.05)

*FMS* fibromyalgia syndrome, *GCPS* graded chronic pain scale

^a^Dependent variable: FMS impact in life

## Discussion

The aim of our study was to assess the relevance of religiosity and spirituality on health outcome in a patient cohort suffering from chronic pain. Our cohort of 102 patients seemed to be less religious than expected, at least when considering religiosity in the traditional way (regarding religious institutions and traditional practices) (Fig. 1). However, spirituality, in the way it was modelled here, was a frequent finding, and most participants believed in a higher existence (supplementary Table 2). The reason for the low traditional religiosity might be the setting of our study in the more secular region of Western Europe compared to, e.g., Latin America or Africa. The world values survey association published a report in 2014 on the degrees of secularization where the percentage of people that consider themselves religious varied widely from 12.9% in China to 99.8% in Pakistan and 95.8% in Nigeria (Inglehart et al. 2010—2014). Another study analyzed religious coping in 54 women in Tanzania with the diagnosis of an obstetric fistula for 14.9 years, comparable to the median disease duration of 14.7 years in our cohort (Table 1) (Watt et al. 2014). 48.1% of these women considered themselves as very religious, 40.7% moderately religious and 0% not religious. These reports confirm that the grade, definition and the performance of religiosity is dependent on the region where patients live.

**Fig. 2:**
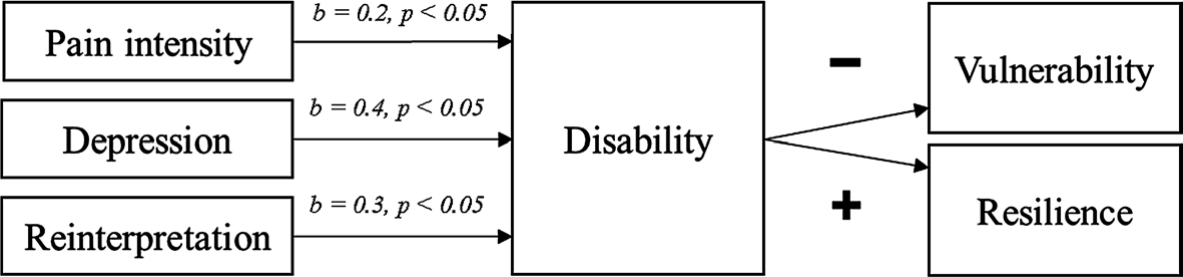
Summary model of disability and resilience. B values present the individual contribution of each predictor to the model and the relationship between life quality and each parameter. Depression has the highest impact on disability (*b* = 0.427, *P* < 0.05). The higher the b value of depression the higher is the resulting disability in life with effects which could result in resilience or vulnerability. Besides depression, the active coping strategy “reinterpretation” and pain intensity have a significant impact on disability

The relevance of religiosity to coping strategies in chronic pain was another aspect that was examined in our study. The correlation tests demonstrated a significant relationship between the dimension “religious orientation” with the coping “praying hoping” (Table 2). However, this might be a chance association, since the regression analysis demonstrated no significant impact of any ASP dimension neither on physical, mental symptoms nor on a specific coping strategy (Table 4). An aspect related to the lower importance of religiosity on coping with FMS could be that chronic pain patients are not confronted to a lethal disease such as cancer. One study analyzed spiritual needs of patients suffering from cancer compared to patients with chronic back pain using the Spiritual Needs Questionnaire (SpRQ) and showed that patients with cancer had higher scores in the SpRQ than chronic pain patients (Büssing et al. 2010). Moreover, as shown by a comparative study on patients with chronic pain in patients with breast cancer, it may be the initial stress of being diagnosed with a serious disease that arouses religious and spiritual needs (Appel et al. 2010). Furthermore, we should have in mind that religious coping is only considered in a few items of the CSQ, which might not be enough to evaluate multidimensional religious aspects (Verra et al. 2006). Nevertheless, the connection between at least one coping type, especially “praying and hoping” shows that a small part of our cohort uses an element of religion to cope with their symptoms. We also see some indirect religious coping in our FMS cohort. The coping strategy “reinterpretation” belongs to spiritual and religious coping strategies and may be positive as well as negative (Pargament et al. 2000). In our cohort, reinterpretation significantly contributed to disability (Fig. 2). Furthermore, patients who reacted aversively to questions about religiosity during the interview sessions, often added comments like “why should God exist when I suffer from pain all day long”. This indicates that religion is not only a positive source of strength (Klein and Berth 2011; Pargament 1997), but doubts and the feeling of being abandoned by God are closely associated to the personal journey to God (Gauthier et al. 2006). Thus, negative connotations of religiosity may also influence the success of coping strategies.

The final purpose of this study was to assess the influence of psychological, physical, spiritual and demographic variables on FMS-related disability. The b-values of the hierarchical regression analysis we applied on 99 valid patient data represent the individual contribution of each predictor to the model and the relationship between each parameter and FMS-related disability. We propose the following model (Fig. 2): Depression, coping “reinterpretation” and pain intensity had a significant impact on FMS-related disability. Depending on the grade of disability, and in combination with other factors like personal characteristics (i.e., sense in life, ability to accept), coping with stress and experienced life events, these three factors may increase or decrease either resilience or vulnerability. Previous studies demonstrated that among other factors active problem- and emotion-focused coping (e.g., reinterpretation) problem solving ability, acceptance and optimism leads to resilience (Denny and Ochsner 2014; Rutter 1985). As expected, and confirming previous data (White et al. 1999), pain intensity had a significant effect on disability. If ineffective coping is used, high pain intensity leads to high disability which promotes vulnerability rather than resilience. The same concept is valid for depression and reinterpretation as an active coping strategy. Reinterpretation belongs to the coping tactic reappraisal—an emotion regulatory skill that is a well-defined and an effective strategy to reduce negative affect and perceived stress (Denny and Ochsner 2014). Redefinition itself is a type of religious coping strategy both positive (benevolent religious reappraisal) and negative (demonic or punishing religious reappraisal) (Pargament et al. 2000). Redefinition is assumed to be effective in pain control, but patients need instructions by a psychologist to use it as a coping for mental and physical symptoms (Denny and Ochsner 2014).We suggest that trained by a coach, patients might achieve a positive effect on their symptoms, resulting in less disability in their daily activities with a positive effect on resilience. They may cope more effectively, adapt to the situation, and may be less vulnerable in stressful situations (Rutter 1985).

## Conclusion

Our patient cohort was moderately religious and disconnected or consciously aversive to classic religious symbols and the church, which might be signs of living in a secular society. The grade of believing in a higher existence plays a role in the choice of coping strategies but has no effects on the outcome of health and mood. Depression, pain catastrophizing and anxiety have a high impact on disability due to FMS, which is highly connected to being resilient or not. Therapies that offer stress-reducing strategies like reappraisal have the potential to increase adapting on stressful situations in the context of pain.

## Supplementary Information

Below is the link to the electronic supplementary material.

Supplementary material 1 (PDF 54 kb)

Supplementary material 2 (PDF 53 kb)

Supplementary material 3 (DOCX 16 kb)
