# Supplementary figures and images for: Relevance of Religiosity for Coping Strategies and Disability in Patients with Fibromyalgia Syndrome
Source: J Relig Health. 2021 Jan 23;61(1):524–39. doi: 10.1007/s10943-020-01177-3 (PMC8837569; doi:10.1007/s10943-020-01177-3)

## Supplementary Figure 1

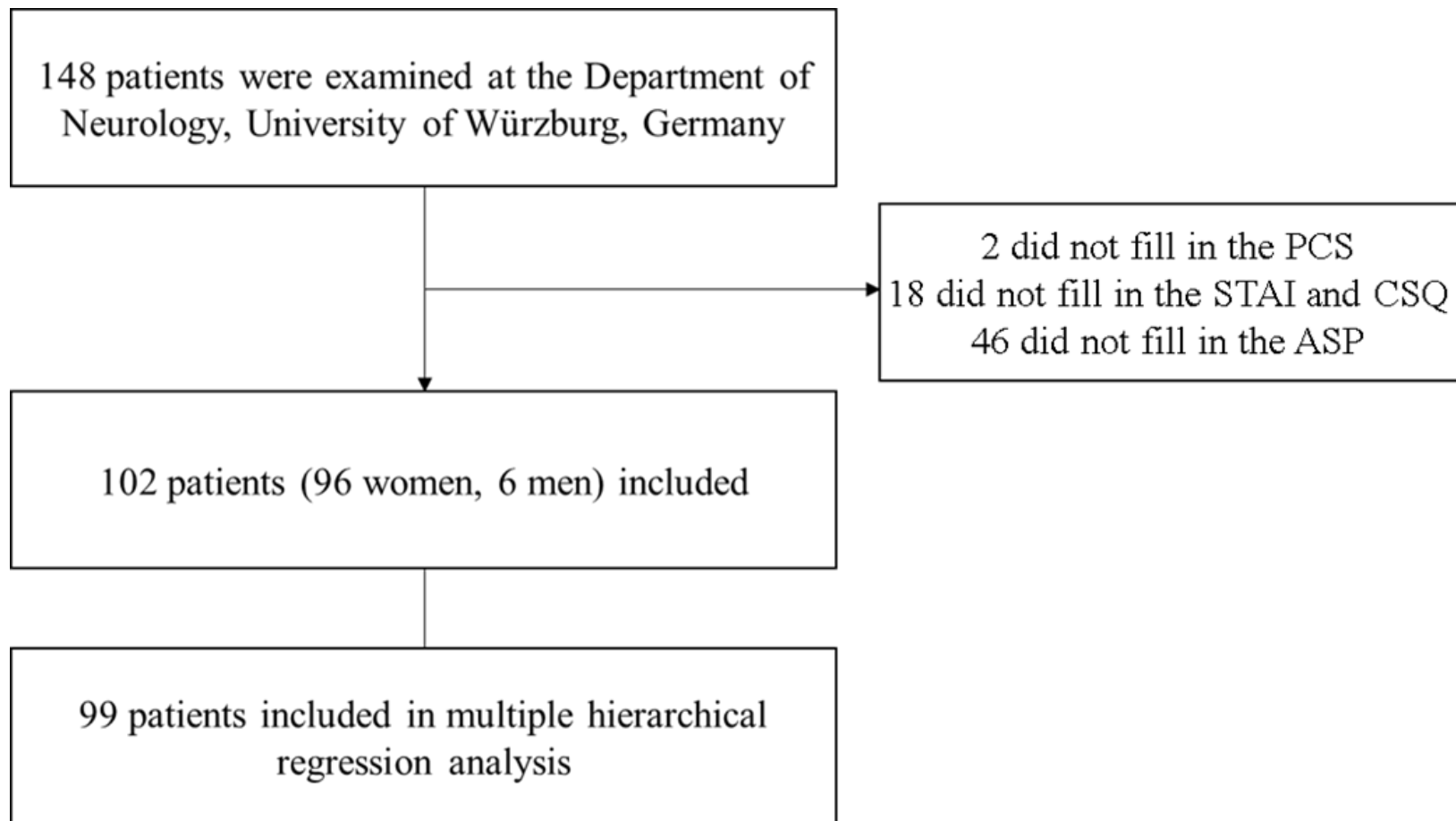

Supplement: Supplementary file 1 — Supplementary material 1 (PDF 54 kb) [file 10943_2020_1177_MOESM1_ESM.pdf]
