# Supplementary material for: Relevance of Religiosity for Coping Strategies and Disability in Patients with Fibromyalgia Syndrome
Source: J Relig Health. 2021 Jan 23;61(1):524–39. doi: 10.1007/s10943-020-01177-3 (PMC8837569; doi:10.1007/s10943-020-01177-3)
Supplement: Supplementary file 2 — Supplementary material 2 (PDF 53 kb) [file 10943_2020_1177_MOESM2_ESM.pdf]

Supplementary Figure 2

Standardized protocol: psychological interview [20 min]

- Good morning Mrs. / Ms., Mr. …. ? How are you? What is bothering you today?
- How do you estimate your coping competence with pain as well as daily problems on a scale from 0 as no competence to 9 best competence?

Part I - childhood and sociality

Evaluation: 2 = burdensome, 0 = neutral, 1 = positive

nr.	Life event item	age [years]	burdensome	neutral	positive
1	serious disease / injury / accident of yourself				
2	serious disease / injury / accident of a close person				
3	death				
4	break up / divorce				
5	serious problem with a good friend / neighbor / relatives (arguments)				
6	unemployness / aimlessness				
7	loss (job / person / theft)				
8	serious financial problems				
9	problems with the police and subpoena				
10	move				
11	intensive care of an old / ill person				
12	abortion / misscarriage / complications				
13	abuse (physical / emotional)				

Part II – faith / belief and values

1. Confession:
2. Independent from your opinion on the church and other religious organizations: Do you believe on a higher existence, that one could name, e.g. God, Jahwe, Allah, higher being, sth. / sb. divine, sth. / sb. absolute?

   ☐ none ☐ low ☐ moderate ☐ high ☐ intense
3. Which of the following terms best characterize your religious attitude?

   ☐ spiritual ☐ religious ☐ atheistic ☐ agnostic ☐ undetermined

   ☐ others: ____________________
4. How important are religiosity and belief in your life at the moment?

   ☐ not ☐ low ☐ moderate ☐ high ☐ intense
5. Which part in your life gives you power, sense and energy?

   ☐ family ☐ religion ☐ sports ☐ nature ☐ friends ☐ job ☐ meditation ☐ activity

Part III – problem solving and learning

Evaluation: 1 = no, 2 = little bit, 3 = high, 4 = intense

Nr.	question	no	little bit	high	intense
1	Do you sometimes think „Why me?“ ?	1	2	3	4
2	Did your disease positively contribute to your personal development?	1	2	3	4
3	Do you ask for help when you have problems?	1	2	3	4
4	Can one solve every problem?	1	2	3	4
5	Can you well take account of your physical and emotional needs?	1	2	3	4
6	Can you maintain these needs towards others?	1	2	3	4
