# Supplementary material for: Relevance of Religiosity for Coping Strategies and Disability in Patients with Fibromyalgia Syndrome
Source: J Relig Health. 2021 Jan 23;61(1):524–39. doi: 10.1007/s10943-020-01177-3 (PMC8837569; doi:10.1007/s10943-020-01177-3)
Supplement: Supplementary file 3 — Supplementary material 3 (DOCX 16 kb) [file 10943_2020_1177_MOESM3_ESM.docx]

**Supplementary Tables**

Supp. Table 1: Characterization of the religious preferences in a subgroup of patients.

|  | **N^*^** | **%** |
| --- | --- | --- |
| **Sample size** | 42 | 100 |
|  |  |  |
| **Spiritual** | 5 | 3.4 |
| **Religious** | 9 | 6.1 |
| **Atheistic** | 4 | 2.7 |
| **Agnostic** | 1 | 0.7 |
| **Not determined** | 1 | 0.7 |
| **Other** | 22 | 14.9 |

^*^N: number

Supp. Table 2: Count of patients related to the grade of believing in a higher existence (interview data, N = 42).

| **Grade of believing in a higher existence** | **Count [N]** |
| --- | --- |
| None | 10 |
| Low | 5 |
| Moderate | 6 |
| High | 15 |
| Intense | 6 |
| **Total** | **42** |

Supp. Table 3: Descriptive statistics of the predictive variables used for the regression analysis.

| **Model** | **Categories** | **Predictor variables** | **M** | **SD** |
| --- | --- | --- | --- | --- |
| **1** | **Demographic variables** | Age | 50.9 | 9.4 |
|  |  | Weight | 75.0 | 14.6 |
|  |  | Height | 166.5 | 7.7 |
|  |  | BMI | 25.4 | 4.9 |
|  |  | Pain duration | 14.1 | 10.1 |
| **2** | **Pain variables** | Neuropathic pain | 0.4 | 0.2 |
|  |  | Pain intensity | 67.2 | 11.8 |
|  |  | GCPS grade | 1.8 | 0.7 |
| **3** | **Mental variables** | Pain catastrophizing | 22.1 | 10.8 |
|  |  | Depression | 23.3 | 11.1 |
|  |  | State anxiety | 47.6 | 13.1 |
|  |  | Trait anxiety | 48.4 | 11.9 |
| **4** | **Coping strategies** | Distraction | 15.5 | 7.0 |
|  |  | Reinterpretation | 6.6 | 6.6 |
|  |  | Self-instructions | 21.1 | 6.8 |
|  |  | Ignore | 15.5 | 7.1 |
|  |  | Praying hoping | 10.0 | 5.7 |
|  |  | Catastrophizing | 17.1 | 7.8 |
|  |  | Activity increase | 18.7 | 5.4 |
|  |  | Pain behavior | 19.9 | 5.4 |
| **5** | **Dimensions of religiosity** | Religious orientation | 36.2 | 25.8 |
|  |  | Search insight / wisdom | 49.7 | 24.3 |
|  |  | Conscious interactions | 75.8 | 14.7 |
|  |  | Transcendence conviction | 47.7 | 25.0 |

SD: standard deviation; M: mean

Supp. Table 4: The ANOVA presents the accuracy of the regression model and the improvement due to the model.

| **Model^a^** |  | **Sum of squares** | **df** | **Mean square** | **F** | **Significance** |
| --- | --- | --- | --- | --- | --- | --- |
| **1** | Regression | 323.5 | 4 | 80.9 | 0.7 | 0.6 |
|  | Residual | 11526.5 | 94 | 122.6 |  |  |
|  | Total | 11850.0 | 98 |  |  |  |
| **2** | Regression | 4.550.6 | 7 | 650.1 | 8.1 | 0.0 |
|  | Residual | 7.299.4 | 91 | 80.2 |  |  |
|  | Total | 11.850.0 | 98 |  |  |  |
| **3** | Regression | 7.303.9 | 11 | 664.0 | 12.7 | 0.0 |
|  | Residual | 4.546.1 | 87 | 52.3 |  |  |
|  | Total | 11.850.0 | 98 |  |  |  |
| **4** | Regression | 7.824.3 | 21 | 372.6 | 7.1 | 0.0 |
|  | Residual | 4.025.7 | 77 | 52.3 |  |  |
|  | Total | 11.850.0 | 98 |  |  |  |
| **5** | Regression | 7.954.2 | 25 | 318.2 | 6.0 | 0.0 |
|  | Residual | 3.895.8 | 73 | 53.4 |  |  |
|  | Total | 11.850.0 | 98 |  |  |  |

^a^ = dependent variable: FMS impact in life; df: degree of freedom; F: F - value
